# Knowledge, awareness and preventive practices of dengue outbreak in Bangladesh: A countrywide study

**DOI:** 10.1371/journal.pone.0252852

**Published:** 2021-06-10

**Authors:** Md. Imam Hossain, Nur E. Alam, Sumaiya Akter, Umme Suriea, Salma Aktar, Siratul Kubra Shifat, Md. Muzahidul Islam, Ihsan Aziz, Md. Muzahidul Islam, Md. Shariful Islam, A. K. M. Mohiuddin

**Affiliations:** 1 Department of Biotechnology and Genetic Engineering, Mawlana Bhashani Science and Technology University, Tangail, Bangladesh; 2 Department of Genetic Engineering and Biotechnology, Jashore University of Science and Technology, Jashore, Bangladesh; 3 Department of Reproductive and Developmental Biology, Graduate School of Life Science, Hokkaido University, Sapporo, Japan; College of Medicine and Sagore Dutta Hospital, INDIA

## Abstract

**Background:**

Dengue, the mosquito borne disease has become a growing public health threat in Bangladesh due to its gradual increasing morbidity and mortality since 2000. In 2019, the country witnessed the worst ever dengue outbreak. The present study was conducted to characterize the socio-economic factors and knowledge, attitude and practice (KAP) status towards dengue among the people of Bangladesh.

**Method:**

A cross-sectional study was conducted with 1,010 randomly selected respondents from nine different administrative regions of Bangladesh between July and November 2019.

A structured questionnaire was used covering socio-demographic characteristics of the participants including their knowledge, awareness, treatment and practices regarding dengue fever. Factors associated with the knowledge and awareness of dengue were investigated separately, using multivariable logistic regression.

**Results:**

Although majority (93.8%) of the respondents had heard about dengue, however, they had still misconceptions about *Aedes* breeding habitat. Around half of the study population (45.7%) had mistaken belief that *Aedes* can breed in dirty water and 43.1% knew that *Aedes* mosquito usually bites around sunrise and sunset. Fever indication was found in 36.6% of people which is the most common symptom of dengue. Among the socio-demographic variables, the level of education of the respondents was identified as an independent predictor for both knowledge (*p<0*.*05*) and awareness (*p<0*.*05*) of dengue. The preventive practice level was moderately less than the knowledge level though there was a significant association (*p<0*.*05*) existed between knowledge and preventive practices. Our study noted that TV/Radio is an effective predominant source of information about dengue fever.

**Conclusion:**

As dengue is emerging in Bangladesh, there is an urgent need to increase health promotion activities through campaigns for eliminating the misconception and considerable knowledge gaps about dengue.

## Introduction

Dengue, the mosquito borne disease, transmitted by the bites of *Aedes* mosquitoes, primarily *Aedes aegypti* and *Aedes albopictus*, is considered the most prevalent human arboviral infection worldwide [1]. Approximately, 3.8 billion people dwelling in 128 countries are perceived to be in danger of dengue infection. According to the WHO, every year about 20,000 deaths occurred on account of dengue globally [2,3]. The cause of dengue fever (DF) is the infection with any one of the 4 serotypes (DENV-1, 2, 3, and 4) of dengue virus and the DF may appear as fatal disease characterized by dengue hemorrhagic fever (DHF) and dengue shock syndrome (DSS) [4,5].

The first dengue virus infection was found in South-East Asia [6] and about 52% of the people who are at risk of dengue globally live in this part of the world. Bangladesh is situated in South Asia and has become an appropriate habitat for the dengue vector and its transmission [7]. In Bangladesh, the first dengue contagion was detected in 1964 [1]. The sporadic cases and small outbreaks clinically suggest that the dengue occurred across the country from 1964 to 1999 but those were not officially reported [8,9]. In the year 2000, a severe outbreak of dengue occurred in Bangladesh with 93 mortality among 5551 morbidity cases [10]. In subsequent years dengue cases reduced remarkably to as low as 375 cases in 2014. However, in 2016, around 6100 dengue cases have been reported with a DENV-2 outbreak in Bangladesh [11]. Three years later, in 2019, Bangladesh experienced highest annual dengue incidence ever reported with 1,12,000 cases and 129 deaths [12].

The incidence and transmission of dengue is influenced by a variety of factors such as uncontrolled population growth, urbanization, deterioration in waste management systems [13] and lack of effective vector control [14]. Due to inadequate water supply, water storage practice is also regarded as a major contributor to dengue epidemics [14]. Moreover, illiteracy, poverty and social inequalities have been associated with poor dengue management [15].

Since no effective vaccine is currently available to prevent dengue, the only possible mode of prevention is vector control [16]. In a prior study, it was revealed that perception of dengue disease risk was much lower, while knowledge of dengue disease among community members has generally been high [17]. For this, community participation is essential at the ground level [5]. The successful participation largely depends on peoples’ knowledge, awareness and attitude towards this disease [14]. Effective dengue prevention and control is an important concern today in Bangladesh as there is an ongoing challenge to ensure proper treatment and prevention options despite having continued progress in dengue research throughout the world [18,19]. Therefore, the objective of this study was to assess nationwide people’s knowledge, attitude and practices (KAP) on dengue.

We discuss the awareness of dengue in Bangladesh and explore future disease risk. Lacking prevention practices and treatment of the disease are also considered, with a focus on population density, human behavior and socio-demographic context.

## Materials and methods

### Study area

**Fig 1:**
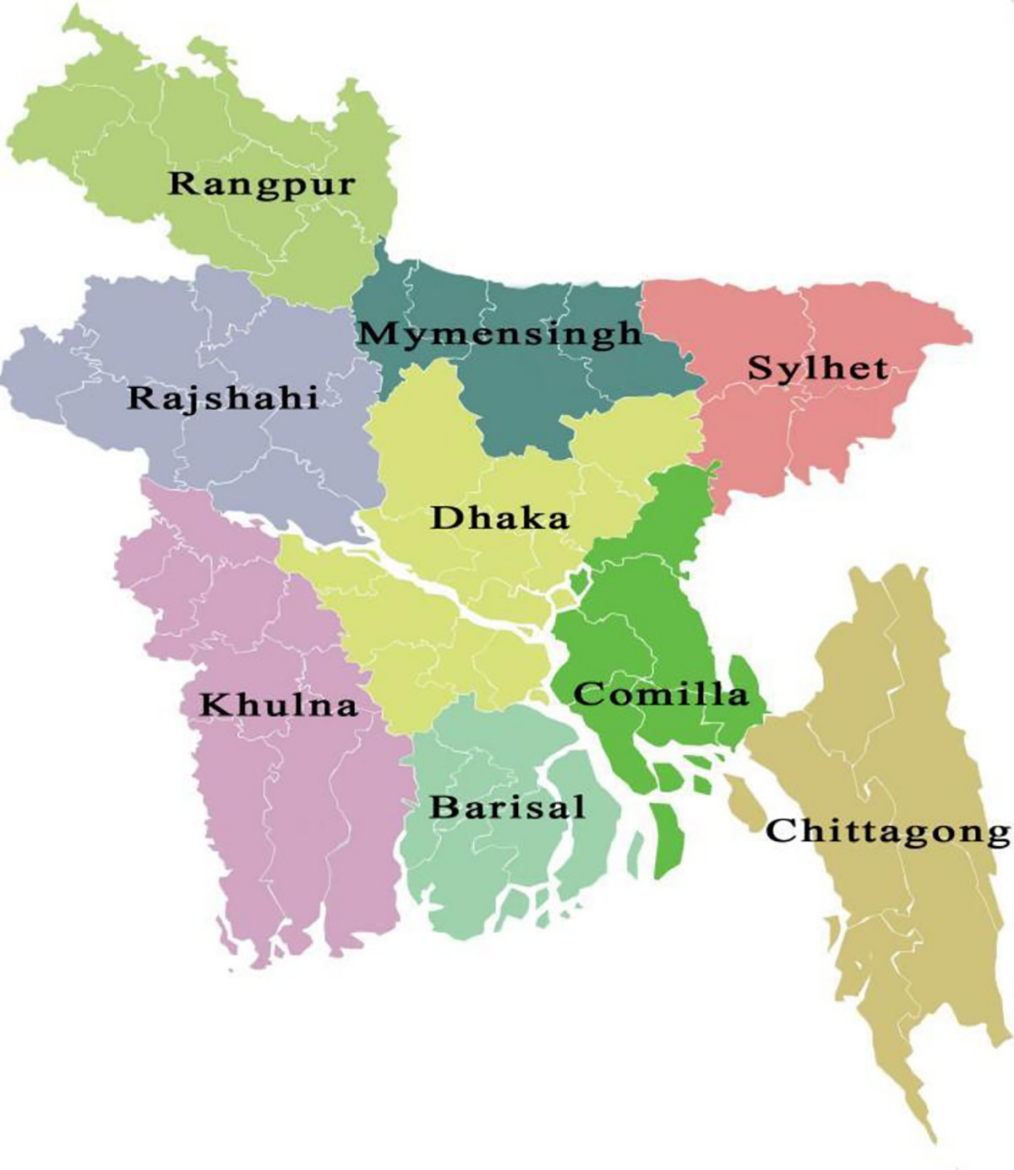
Map of the objective regions of Bangladesh.

In Bangladesh, some previous studies revealed that dengue is getting epidemic in all over the country [1,20–22] as well as dengue risk is very heterogeneous across the country [23]. Regarding those, to assess people’s KAP from all parts of Bangladesh, the study was conducted among nine administrative regions across Bangladesh (**Fig 1**). We selected a study area based on population density from every specific region except Dhaka. Dhaka is the biggest city of Bangladesh as it had a population of 11.8 million (based on 2011 census) and a study showed that Dhaka is the highest risk prone area for dengue transmission in Bangladesh [24]. Therefore, we selected two area named Dhaka metropolitan and Tangail from Dhaka region for doubling this regional’s sample size.

### Data collection

A cross-sectional survey was carried out among 14 - >60 (up to 75) years old randomly selected 1,010 peoples in nine administrative regions (Dhaka, Comilla, Chittagong, Barisal, Khulna, Rajshahi, Rangpur, Mymensingh and Sylhet) of Bangladesh between July and November 2019. An assumption of 50% prevalence of good KAP and an absolute precision of 5% were taken for calculating the minimal sample size which was found 385. This was then multiplied by the design effect of 2.55, as we used cluster sampling technique. The sample size was further increased by 3% to allow for non-sampling error, particularly nonresponse error. Thus, the final estimated sample size was 1,010 which was considered adequate to fulfill the objectives of our study at a 95% confidence level. Respondents were selected from different places, such as houses, local market and public institution in order to collect data from peoples of various backgrounds in the community. Respondents were approached by interviewers at various times of the day during data collection. People who were failed to respond to all questions and who left before completing the interview were excluded. With the exception of Dhaka and Dhaka region, data were collected from 100 peoples per region totaling 800 data points for each question were collected from eight regions. For Dhaka region, a total of 210 samples were collected, 100 from Dhaka metropolitan area and remaining 110 from outside of Dhaka metropolitan area i.e. Tangail.

### Questionnaire content

The questionnaire was developed by our research team according to the literature of the previous peer-reviewed studies [14,25–27]. The study questionnaire was developed in English, then verbally translated into local language i.e. Bengali by the interviewer during the interview time.

The questionnaire was divided into five sections. The first section consisted of 10 questions that covered the socio-demographic profile of the respondents. The second section also consisted of 10 questions that covered the knowledge of dengue fever. The section three, on the other hand, had five questions that covered the awareness on dengue fever. The section four consisted of six questions on knowledge and practices of dengue fever treatment. The last section covered 8 questions on knowledge and awareness regarding dengue prevention. The type of questions in the five sections were consisted of either closed-ended questions with ‘yes’, ‘no’, ‘don’t know’, or multiple-choice questions. Before conducting the actual data collection, the questionnaire was pilot tested in a community with similarities to the study population. Knowledge score was assessed on ten essential questions. According to our criteria, for ‘‘sufficient knowledge”, the respondent needed to have correct responses to at least six questions. Less than six correct responses were termed ‘‘insufficient knowledge”.

Pearson’s correlation was used to analyze the construct validity of the questionnaire. The correlation matrix demonstrated the existing relationships between the pairs of variables. The analysis revealed that all of the 37 questions of the questionnaire were significantly correlated except family income (Q-9) which was not considered for any association.

Cronbach’s Alpha was used to assess the reliability coefficient which is a measure of the internal consistency of the questionnaire. The Cronbach’s alpha coefficient for KAP questions was 0.623 where the value between 0.6–0.7 indicates an acceptable level of reliability [28].

### Statistical analysis

Each filled questionnaire was checked, and coded individually at the end of interview. Care was taken to confirm the accuracy and uniformity of the data. The data collected from the survey were analyzed by IBM SPSS version 20. Descriptive statistics such as frequency and percentages were calculated for categorical variables (e.g. gender, living place), while mean and standard deviation were calculated for continuous variable “age” in the current study with their respective 95% confidence intervals. Socio-demographic characteristics (e.g. employment status, gender, literacy status) had been considered as indicator variables while KAP (e.g. mode of spread, preventive measures, common symptoms) as outcome variables. Multivariable logistic regression models were generated to assess factors associated with knowledge and awareness of dengue fever. Adjusted odds ratios (aORs) and its 95% confidence intervals (CIs) were estimated. First, variables of interest were assessed using univariate analysis. Any factor that provided a univariate *p*-value ≤ 0.25 was entered into the multivariate analysis. The following variables were adjusted for in the models: gender, age, living place, literacy, employment and socio-economic status. Collinearity was assessed using the variance inflation factor (VIF) to ensure a strong linear relationship among independent variables included in the model was not present. The goodness of fit of the model was checked using the Hosmer Lemeshow (H-L) test. The association between aware and preventing ways of dengue with knowledge of dengue fever were measured using chi-square test. P-values of *<* 0.05 were considered significant. Microsoft Excel version 2016 was used to draw graphs wherever appropriate.

### Ethical clearance

The study was approved by the Faculty of Life Science of Mawlana Bhashani Science and Technology University, Tangail-1902, Bangladesh (MBSTU/L.S.F./Re.Let./67/41(B)). The respondents were given an explanation of the objectives and benefits of the study. Before the interview, verbal consent was taken from the respondents according to the WHO and Bangladesh Medical Research Council (BMRC) guidelines of ethical consideration. Extra verbal consent was also obtained from guardians for house respondents in case of under the legal age. Respondent’s right to refuse and withdraw from study any time was accepted. Confidentiality of the respondents was maintained.

## Results

### Socio-demographic characteristics

A total of 1100 individuals were approached, only 1010 were interviewed completely, giving a response rate of 91.82%. The profile of the respondents is given in **Table 1** that summarizes the socio-demographic characteristics. There were 506 male (50.1%) and 504 female (49.9%) respondents among the 1010 responders and the majority (54.6%; 551/1010) were 16 to 30 years old. Mean age of respondents was 31.96 ± 13.8 years (age range: 14–75 years). Fifty four percent of the respondents were from rural community (village) whereas 26.6% and 19.4% were from semi-town and city, respectively. About 12.6% of the respondents were illiterate and nearly 18% were graduates. Of the total respondents, 34.4% were students; 21.1% were housewives and 40.7% had monthly family income less than 15,000 BDT. Approximately 16% peoples were from lower-income family and almost 47% were from middle-income family among the respondents.

**Table 1:** Socio-demographic characteristics of the respondents in Bangladesh.

Characteristics	n (%)
**Gender**	
Male	506 (50.1)
Female	504 (49.9)
**Age (year)**	
1–15	38 (3.8)
16–30	551 (54.6)
31–45	249 (24.7)
46–60	124 (12.3)
61–75	48 (4.8)
Mean ± SD*	**31.96 ±13.8**
**Living place**	
Village	545 (54.0)
Semi Town	269 (26.6)
City	196 (19.4)
**Literacy status**	
No formal schooling (illiterate)	127 (12.6)
Primary	172 (17.0)
Secondary	258 (25.5)
Intermediate	227 (22.5)
Graduate	180 (17.8)
Post Graduate	46 (4.6)
**Employment status**	
Job holder	88 (8.7)
Teacher	75 (7.4)
Businessman	107 (10.6)
Farmer	53 (5.2)
Student	347 (34.4)
Laborer	49 (4.9)
Housewife	213 (21.1)
Nothing as mentions	38 (3.8)
Others	40 (4.0)
**Family income (Taka/month)**	
<15,000	411 (40.7)
<30,000	344 (34.1)
<50,000	187 (18.5)
>50,000	68 (6.7)
**Socio economic status**	
Lower	163 (16.1)
Lower middle	303 (30.0)
Middle	474 (46.9)
Upper middle	70 (6.9)

* SD = Standard deviation.

### Knowledge, attitude and preventive practice (KAP) towards DF

Among the respondents, 93.8% had heard about dengue fever (DF) and most of them (61.3%) were aware of dengue outbreak in Bangladesh (**Table 2)**. Most (91.3%) believed that it was transmitted through mosquito bites. A small number (2.1%) opined that this disease is transmitted through dirty drinking water.

**Table 2:** Knowledge, attitude and preventive practice (KAP) towards dengue fever in different regions of Bangladesh.

Variables	n (%)	95% CI*
**Heard about dengue fever**		
Yes	947 (93.8)	1.05–1.08
No	63 (6.2)	
**Mode of spread**		
Mosquito’s bite	922 (91.3)	1.21–1.33
Dirty drinking water	21 (2.1)	
Contaminated food	0 (0.0)	
Others	15 (1.5)	
Don’t know	52 (5.1)	
**Carrier of dengue fever**		
Aedes	609 (60.3)	2.0–2.17
Anopheles	27 (2.7)	
All types of mosquito	55 (5.4)	
Don’t know	319 (31.6)	
**Most frequent mosquito biting time**		
Sunrise/sunset	435 (43.1)	1.93–2.06
Night	301 (29.8)	
Afternoon	120 (11.9)	
Don’t know	154 (15.2)	
**Dengue is transmissible**		
Yes	512 (50.7)	1.46–1.52
No	498 (49.3)	
**Dengue is transmitted through**		
Human to human contact	62 (6.1)	3.72–3.91
Blood transfusion	289 (28.6)	
Needle stick injury	25 (2.5)	
Sharing of food/ clothes with the patient	35 (3.5)	
Don’t know	599 (59.3)	
**Common breeding site**		
In clean water	434 (43.0)	1.64–1.72
In unclean water	462 (45.7)	
Don’t know	114 (11.3)	
**Common symptoms**		
High fever	370 (36.6)	3.95–4.31
Severe body aches	74 (7.3)	
Nausea and vomiting	81 (8.0)	
Red spots on the body	56 (5.5)	
Diarrhea	12 (1.2)	
Pain abdomen	8 (0.8)	
All of the above	195 (19.3)	
Don’t know	214 (21.2)	
**Awareness about dengue fever**		
Yes	619 (61.3)	1.36–1.42
No	391 (38.7)	
**Preventive measures**		
Use mosquito spray	92 (9.1)	3.76–4.05
Use mosquito coil	389 (38.5)	
Use mosquito repellant/cream	89 (8.8)	
Keep closed windows & doors	54 (5.3)	
Use smoke to drive away mosquitoes	10 (1.0)	
Keep neat & clean surroundings	131 (13.0)	
Use mosquito net	223 (22.1)	
Cover body with long clothes	4 (0.4)	
Do nothing	18 (1.8)	

*CI = Confidence Interval.

Approximately, two-third of the respondents could correctly answer that *Aedes* mosquito spread dengue virus. The majority of the respondents (45.7%) had misconceptions that unclean water-holding containers could be potential breeding places for *Aedes* mosquito. Less than half of the respondents (43.1%) knew that *Aedes* mosquito usually bites during sunrise/sunset.

Fever was the most consistent response (36.6%) when asked about the common symptoms of dengue. About half (50.7%) of the respondents believed that dengue could be transmitted and 1/3 (289; 28.6%) opined that it could be transmitted through blood transfusion.

Dengue fever can be avoided by using preventive measures. For personal-protective measures, 38.5% and 22.1% were responded that mosquito coils and mosquito nets were considered the most effective options for prevention while some (8.8%) were used mosquito repellant/cream as an alternative **(Table 2**).

**Fig 2:**
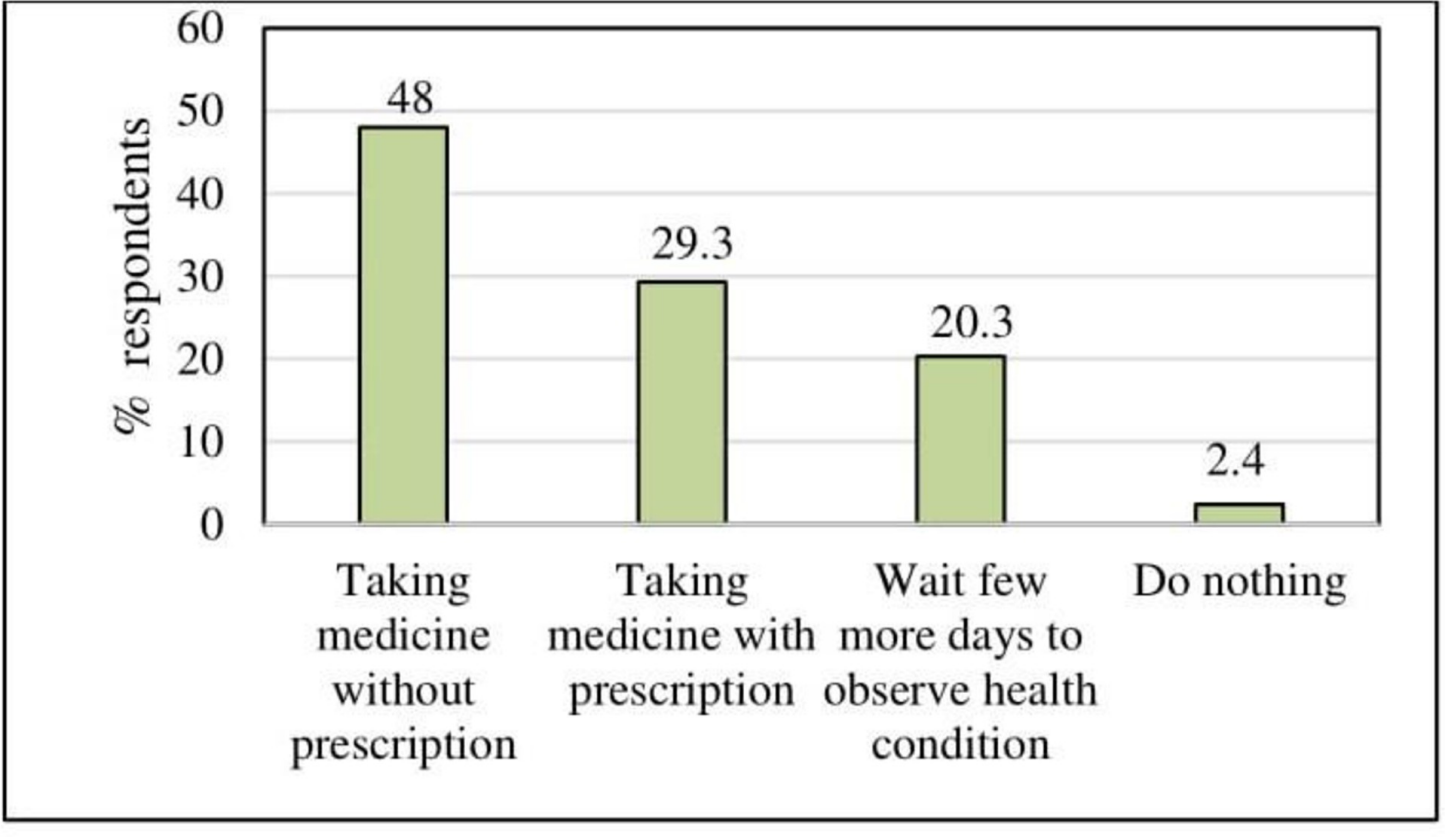
Steps after noticing any symptoms of fever.

In Bangladesh, treatment seeking behavior is impacted by financial status. Usually, a large number of people after affected by any diseases purchase over the counter medication from nearby pharmacy without any prescription. About 48% (485/1010) respondents answered to take medicine without prescription followed by 29.3% (296/1010) took medicine with prescription after noticing symptoms of fever (**Fig 2**).

When respondents were asked about the treatment, majority (89.6%) answered dengue fever was treatable but most of them (59.3%) were not aware of the primary treatment (pre-medical like bed rest, drinking plenty of fluids). Only 21.8% of people did test immediately after suffering from fever and 38.3% were symptomless (without fever) (**Table 3**).

**Table 3:** Knowledge and attitude about dengue fever treatment in Bangladesh.

Variables	n (%)	95% CI
**Dengue fever is treatable**		
Yes	905 (89.6)	1.12–1.18
No	59 (5.8)	
Don’t know	46 (4.6)	
**Knowing primary (pre-medical) treatment of Dengue fever**		
Yes	411 (40.7)	1.56–1.62
No	599 (59.3)	
**Knowledge about which tests are required to diagnose**		
Yes	308 (30.5)	1.67–1.72
No	702 (69.5)	
**Test after suffering from fever**		
Immediately	220(21.8)	3.39–3.58
After getting serious condition	67(6.6)	
After a few days	110 (10.9)	
Never do test	226 (22.4)	
Didn’t face dengue yet	387 (38.3)	

### Source of information on dengue fever

**Fig 3:**
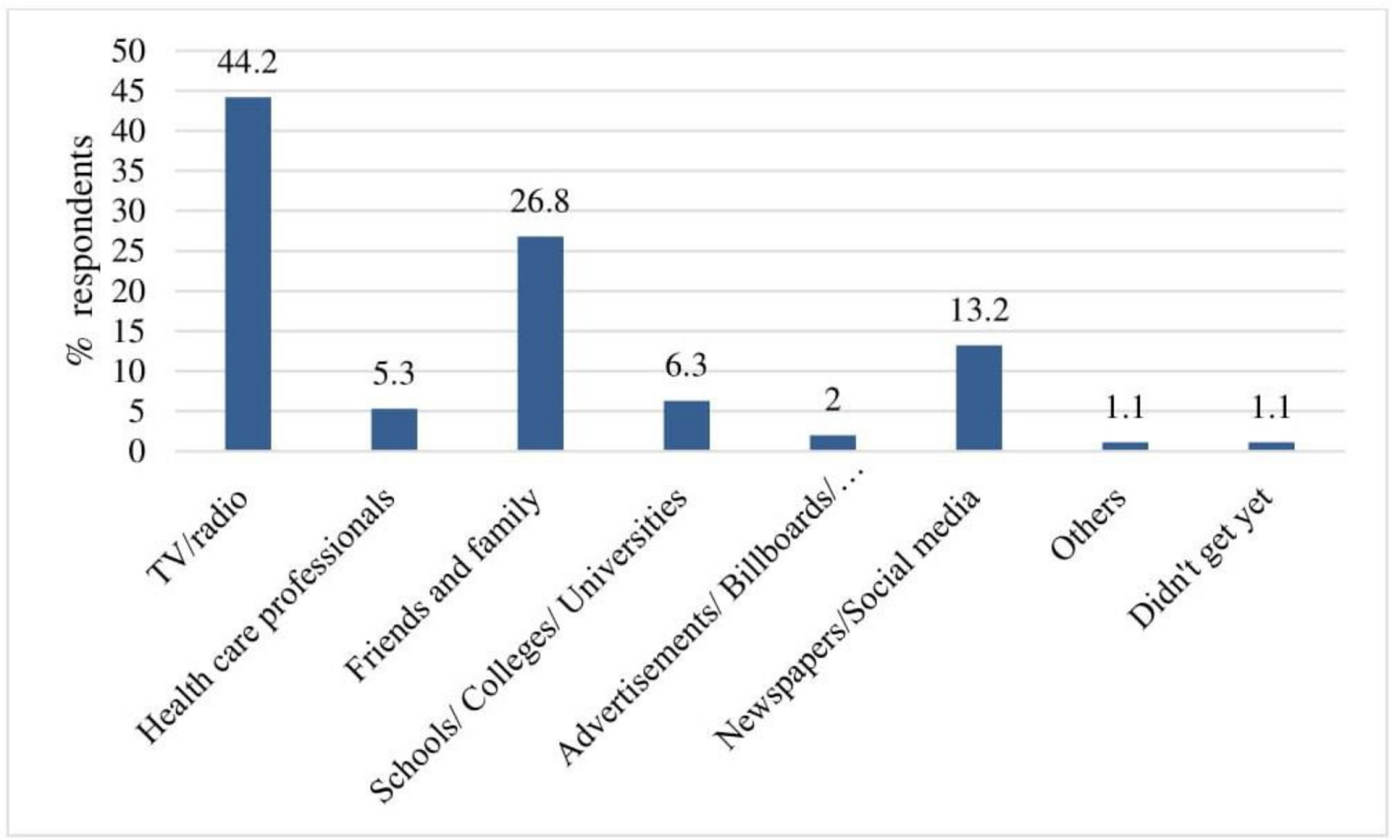
Source of information in dengue fever in Bangladesh.

In this study, 44.2% respondents identified television/radio was the leading source of information about dengue fever followed by friends and family (26.8%). On the other hand, 13.2% stated that newspapers/social media were source of information on dengue and about 6.3% of respondents had obtained knowledge through different educational institutions (**Fig 3**).

### Association among knowledge, awareness, and practice about dengue

About 65.9% of respondents had sufficient knowledge about dengue. The respondents who lived in the city were more knowledgeable (adjusted odds ratio [aOR]: 1.097; 95% confidence interval [CI]: 0.635–1.897) than those who were lived in villages/rural areas and semi town in Bangladesh. Respondents with a qualification of graduate (aOR: 0.997; 95% CI: 0.356–2.789) or post-graduate (aOR: 0.717; 95% CI: 0.250–2.058) were more than 3 times more knowledgeable than those who did not have formal schooling. Moreover, lower and lower middle economic respondents were less informed than middle (aOR: 0.872; 95% CI: 0.425–1.791) and upper middle (aOR: 0.788; 95% CI: 0.395–1.570) economic respondents. However, respondents’ knowledge score on dengue varied significantly by the area of residence (*p* = 0.030), level of education (*p*<0.001), and socio-economic status (*p* = 0.026) (**Table 4**). The H-L p value for the model was 0.522.

**Table 4:** Univariate and multivariable analyses of factors associated with knowledge of dengue (n = 1010).

Variables	Knowledge on dengue		Univariate		Multivariate	
	Sufficient n (%)	Insufficient n (%)	OR (95% CI)	*p* value	OR (95% CI)	*p* value
**Gender**						
Male	318 (31.5)	188 (18.6)	Ref.	0.044	Ref.	0.295
Female	347 (34.4)	157 (15.5)	1.307 (1.007–1.696)		1.240 (0.829–1.857)	
**Age (year)**						
1–15	27 (71.1)	11 (28.9)	Ref.	<0.001	Ref.	0.063
16–30	400 (72.6)	151 (27.4)	0.223 (0.089–0.559)		0.204 (0.070–0.595)	
31–45	153 (61.4)	96 (38.6)	0.207 (0.111–0.385)		0.483 (0.230–1.013)	
46–60	68 (54.8)	56 (45.2)	0.344 (0.181–0.655)		0.497 (0.237–1.043)	
61–75	17 (35.4)	31 (64.6)	0.452 (0.227–0.900)		0.603 (0.273–1.330)	
**Living place**						
Village	301 (55.2)	244 (44.8)	Ref.	<0.001	Ref.	**0.030**
Semi town	200 (74.3)	69 (25.7)	4.154 (2.744–6.290)		1.674 (1.017–2.756)	
City	164 (83.7)	32 (16.3)	1.768 (1.108–2.821)		1.097 (0.635–1.897)	
**Literacy status**						
No formal schooling	29 (22.8)	98 (77.2)	Ref.	<0.001	Ref.	**<0.001**
Primary	76 (44.2)	96 (55.8)	22.529 (8.688–58.420)		9.523 (3.137–28.910)	
Secondary	168 (65.1)	90 (34.9)	8.421 (3.392–20.906)		5.306 (1.845–15.258)	
Intermediate	191 (84.1)	36 (15.9)	3.571 (1.459–8.744)		2.345 (0.840–6.548)	
Graduate	161 (89.4)	19 (10.6)	1.257 (0.496–3.182)		0.997 (0.356–2.789)	
Post-graduate	40 (87)	6 (13)	0.787 (0.295–2.098)		0.717 (0.250–2.058)	
**Employment status**						
Job holder	68 (77.3)	20 (22.7)	Ref.	<0.001	Ref.	0.510
Teacher	68 (90.7)	7 (9.3)	0.266 (0.120–0.590)		0.969 (0.383–2.450)	
Businessman	73 (68.2)	34 (31.8)	0.093 (0.034–0.252)		0.434 (0.140–1.342)	
Farmer	15 (28.3)	38 (71.7)	0.421 (0.201–0.885)		0.755 (0.322–1.772)	
Student	272 (78.4)	75 (21.6)	2.292 (0.968–5.425)		1.285 (0.491–3.362)	
Laborer	12 (24.5)	37 (75.5)	0.249 (0.128–0.488)		0.965 (0.423–2.202)	
Housewife	120 (56.3)	93 (43.7)	2.790 (1.135–6.858)		1.576 (0.583–4.256)	
Nothing as mention	18 (47.4)	20 (52.6)	0.701 (0.356–1.380)		1.079 (0.463–2.515)	
Others	19 (47.5)	21 (52.5)	1.005 (0.413–2.446)		1.115 (0.399–3.114)	
**Socio economic status**						
Lower	74 (45.4)	89 (54.6)	Ref.	<0.001	Ref.	**0.026**
Lower middle	182 (60.1)	121 (39.9)	3.750 (2.002–7.021)		1.599 (0.723–3.537)	
Middle	356 (75.1)	118 (24.9)	2.073 (1.146–3.749)		0.872 (0.425–1.791)	
Upper middle	53 (75.7)	17 (24.3)	1.033 (0.576–1.854)		0.788 (0.395–1.570)	

Bold shows factors that were significant.

In the multivariable logistic regression model of potential predictors of awareness on dengue fever, participants were more aware when they were graduate (aOR: 0.861; 95% CI: 0.402–1.844) and post-graduate (aOR: 0.704; 95% CI: 0.328–1.511). We found a significant association between awareness on dengue fever (Q21) and the education level of the respondents (*p* = 0.003). However, middle (64.6%) and upper middle (72.9%) economic respondents were more conscious compare to lower and lower middle economic respondents, but there was no statistical significance found (**Table 5**). The H-L p value for the model was 0.078. **Table 6** represents the significant positive association of knowledge with preventing ways and preventing practice of *Aedes* reproduction (*p<0*.*05*).

**Table 5:** Univariate and multivariable analyses of factors associated with awareness on dengue fever (Q-21).

Variables	Awareness on dengue		Univariate		Multivariate	
	Yes n (%)	No n (%)	OR (95% CI)	*p* value	OR (95% CI)	*p* value
**Gender**						
Male	305 (60.3)	201 (39.7)	Ref.	0.509	-	-
Female	314 (62.3)	190 (37.7)	1.089 (0.845–1.403)			
**Age (year)**						
1–15	21 (55.3)	17 (44.7)	Ref.	0.251	-	-
16–30	351 (63.7)	200 (36.3)	1.349 (0.567–3.208)			
31–45	151 (60.6)	98 (39.4)	0.950 (0.516–1.747)			
46–60	66 (53.2)	58 (46.8)	1.082 (0.572–2.046)			
61–75	30 (62.5)	18 (37.5)	1.465 (0.740–2.898)			
**Living place**						
Village	331 (60.7)	214 (39.3)	Ref.	**0.030**	Ref.	0.067
Semi town	153 (56.9)	116 (43.1)	1.431 (1.010–2.026)		0.813 (0.542–1.220)	
City	135 (68.9)	61 (31.1)	1.678 (1.140–2.470)		1.194 (0.776–1.837)	
**Literacy status**						
No formal schooling	65 (51.2)	62 (48.8)	Ref.	**<0.001**	Ref.	**0.003**
Primary	87 (50.6)	85 (49.4)	2.180 (1.063–4.470)		1.554 (0.658–3.673)	
Secondary	142 (55)	116 (45)	2.233 (1.114–4.477)		1.682 (0.750–3.769)	
Intermediate	158 (69.6)	69 (30.4)	1.867 (0.951–3.664)		1.586 (0.729–3.451)	
Graduate	135 (75)	62 (25)	0.998 (0.501–1.988)		0.861 (0.402–1.844)	
Post-graduate	32 (69.6)	14 (30.4)	0.762 (0.373–1.554)		0.704 (0.328–1.511)	
**Employment status**						
Job holder	59 (67)	29 (33)	Ref.	**0.001**	Ref.	0.435
Teacher	52 (69.3)	23 (30.7)	0.445 (0.207–0.954)		.576 (0.254–1.305)	
Businessman	70 (65.4)	37 (34.6)	0.400 (0.181–0.883)		.538 (0.225–1.285)	
Farmer	24 (45.3)	29 (54.7)	0.478 (0.229–1)		.528 (0.245–1.139)	
Student	231 (66.6)	116 (33.4)	1.093 (0.480–2.490)		1.018 (0.433–2.394)	
Laborer	20 (40.8)	29 (59.2)	0.454 (0.235–0.879)		.590 (0.291–1.195)	
Housewife	121 (56.8)	92 (43.2)	1.312 (0.565–3.046)		1.113 (0.467–2.653)	
Nothing as mention	23 (60.5)	15 (39.5)	0.688 (0.349–1.354)		.693 (0.343–1.399)	
Others	19 (47.5)	21 (52.5)	0.590 (0.240–1.450)		0.529 (0.212–1.321)	
**Socio economic status**						
Lower	91 (55.8)	72 (44.2)	Ref.	**0.012**	Ref.	0.584
Lower middle	171 (56.4)	132 (43.6)	2.124 (1.153–3.912)		1.480 (0.750–2.918)	
Middle	306 (64.6)	168 (35.4)	2.072 (1.168–3.677)		1.546 (0.832–2.872)	
Upper middle	51 (72.9)	19 (27.1)	1.474 (0.842–2.578)		1.390 (0.773–2.499)	

**Table 6:** Association between aware (Q-21), preventing ways (Q-33) and preventing practice (Q-35), and knowledge of dengue fever.

Variable	Chi-square	*p* value	OR (95% CI)
Aware of Dengue fever	16.082	<0.001	1.717 (1.317–2.238)
Preventing ways of Dengue fever	108.947	<0.001	4.174 (3.168–5.497)
Preventing practice of *Aedes* reproduction	27.127	< 0.001	2.098 (1.583–2.780)

## Discussion

As Bangladesh is situated in the Southeast (SE) Asian region, vector-borne diseases like dengue, are perceived as a major health threat [10]. Overpopulation, impromptu and uncontrolled urbanization are identified as the key factors for increasing transmission of mosquito-borne diseases like dengue [1]. Socio-demographic factors and KAPs among the population play a critical role in both incidence of dengue epidemics and implementation of control measures [16]. To control the dengue virus, it was previously revealed that lack of knowledge about clinical features or control measures is the most common problem [29]. This study was conducted to assess the dengue knowledge, attitudes, and practices of people throughout the Bangladesh.

In the present study, nearly most of the respondents heard of dengue though many of them do not have still the basic knowledge on dengue. For example, there were still misconceptions about the breeding sites of the dengue vector. Many of the respondents stated the dirty and unclean water such as sewage drains are the most common breeding sites for dengue mosquitoes. The knowledge level on DF found in our study is comparable to similar KAP study findings previously conducted in the Dhaka city of Bangladesh [20], India [30], Pakistan [25], Malaysia [5], Thailand [31], Brazil [32] and Jamaica [26]. In this study, some of the respondents were able to correctly identify all of the symptoms of DF compared to many respondents who identified fever as an obvious symptom. Most of the respondents belong to this study had not personally experienced with dengue fever nor any of their family member infected with this disease, that’s why they didn’t state the other typical symptoms of DF. Among the study population with poor knowledge of symptoms associated with DF, the disease might easily be confused with other common causes of fever such as influenza and typhoid [26] even COVID-19.

A large portion of respondents in this study mentioned that dengue spreads through mosquito bites which is similar to other studies done in India [33], Malaysia [34] and Brazil [32], but only 60.3% of the respondents stated that *Aedes* mosquitoes transmit dengue virus (DENV). The dengue vector *A*. *aegypti* bite mostly during several hours after dawn and before dusk [26]. More importantly, 43.1% of respondents reported that mosquitoes bite during the period of sunrise/sunset. This finding was similar to some previous studies which were performed in different countries where majority of respondents knew that dengue vectors might bite at sunrise or sunset [32,35].

In this study, 28.6% of the respondents stated that the dengue virus could be contracted through blood transfusion. However, in reality, during blood or organ transplant the contraction of virus is in rare [36]. For example, an estimated risk was reported for dengue-infected blood transfusions to be 1.625–6/10,000 in Singapore in 2005 [37].

A large number of the respondents (44.2%) in this study indicated that television/radio was the most common source of DF information. Similar findings were reported in previous studies from India [38], Indonesia [39], Nepal [40], Laos [41] and the Philippines [42]. This indicated that the mass media have an impact to convey health information more rapidly to the general people in Bangladesh. A study from Laos where friends or relatives were the major source of information regarding dengue fever [43]. Health personnel were also mentioned as a source of dengue fever information in a study from Thailand [44].

Most of the respondents in the current study (89.6%) knew that dengue fever is treatable. More than 40% people agreed that they had known about the primary treatment like get plenty of bed rest and drinking of lots of water. Similar finding was reported in a study from Jamaica [26]. In this study, around one-third of the respondents reported to know about the tests normally required to diagnose dengue and 21.8% respondents stated that they perform these tests immediately after suffering from fever to confirm dengue.

The mostly favored preventive measures used by the respondents reported in this study was using of mosquito coils (38.5%). Using bed nets would be ineffective in preventing *Aedes* mosquito bites as most of them were used only at night [30]. Only a few of the participants (13%) responded that cleaning the surroundings can be preventive measures. This finding was incongruous with a previous study where more than half (55.7%) of the respondents mentioned that cleaning of the surroundings as an important preventive practice [33]. In the study setting, though majority of the respondents had moderate knowledge about *Aedes* mosquito and dengue fever, very few respondents adopted practices for preventing and controlling *Aedes* mosquito. The good practice level was less than the knowledge level though there was significant association existing between knowledge and practice. Thus, poor practices along with a high density of the human population, and a suitable environment for the reproduction of *Aedes* in Bangladesh more specifically in major cities might contribute to the greater risk of transmission. These findings on practice levels are similar to those of other studies which reported high levels of knowledge but low levels of practice [45,46]. In fact, if the gap between knowledge and practice isn’t decreased, then it will be an important challenge for controlling dengue and *Aedes* populations.

This study found that level of education, residence (living place) and socio-economic status were significantly associated with the knowledge scores of the respondents. The associations between literacy level and knowledge about dengue had also been shown in another study [25] which indicates that the higher the education level, the better the knowledge on dengue.

Based on our study, it is recommended that public education on dengue should be emphasized for successful dengue control program [47]. Effective education programs, public health campaigns by local NGOs and the Ministry of health should be performed especially in rural and densely populated area specifically about dengue transmission, *Aedes* mosquitoes breeding sites, early dengue diagnosis and treatment procedure. Adequate health personnel should be provided by the Ministry of health and trained them to give appropriate counseling in an effort to bring about behavioral changes among the population for promoting prevention practice of dengue. Moreover, continuous education and monitoring should be done to ensure long-term behavioral changes towards successful dengue prevention.

### Strength and limitation

To our knowledge, this is the first study that aims to assess the public knowledge, attitudes and preventive practices towards dengue throughout the country where dengue is a growing concern. Findings from our study will serve as guides to health care planners for better strategic planning of dengue control. KAP studies will provide a suitable format to evaluate existing programs and to identify effective strategies for behavioral change [46]. However, selection of study area and respondents in this study were based on convenience sampling, this might have caused some bias in the representation of our respondents. The possibility of interviewer bias may also be occurred. Furthermore, a cross-sectional survey of this nature may capture only a snapshot of information about the respondents but cannot be generalized to other populations; the findings may change over time. As the survey questionnaire based on multiple choice, it is possible that some respondents might provide socially desirable responses to some questions [48].

## Conclusion

The mosquito–borne disease dengue is emerging in Bangladesh gradually at an alarming rate. In spite of having moderate knowledge of dengue transmission vectors and symptoms, the preventive practices are not well executed. However, it was found that the paucity of basic knowledge on dengue epidemiology and vector bionomics among the population could be a major cause for the increasing trend of dengue in this highly populated country. So, an emphasis should be provided on health education programme especially on dengue disease to increase community knowledge and awareness towards *Aedes* mosquitoes and DF prevention countrywide.

## Supporting information

S1 File Dengue questionnaire. (PDF)

S2 File Dengue SPSS file. (SAV)
