# Supplementary material for: Knowledge, awareness and preventive practices of dengue outbreak in Bangladesh: A countrywide study
Source: PLoS One. 2021 Jun 10;16(6):e0252852. doi: 10.1371/journal.pone.0252852 (PMC8192001; doi:10.1371/journal.pone.0252852)
Supplement: S1 File — (PDF) [file pone.0252852.s001.pdf]

Sample ID: **Form #**

Socio-demographic profile:

1. Name: ……………………………………..
2. Age (Year):
   i. 1 - 15 ii. 16 – 30 iii. 31 – 45 iv. 46 – 60 v. > 60
3. Gender: i. Male ii. Female
4. District: ………………………………
5. Division: ……………………………..
6. Living Place: (i) Village (ii) Semi Town (iii) City
7. Literacy status: i. No formal schooling ii. Primary iii. Secondary
   iv. Intermediate v. Graduate vi. Post-Graduate
8. Employment status: Job holder/ Teacher/ Businessman/ Farmer/ Student/ Laborer/ Housewife/ Nothing as mention/ Others
9. Family Income(Taka)/Month: i. < 15,000 ii. <30,000
   iii. <50,000 iv. >50,000
10. Socio-economic status: Lower class/ Lower middle class/ Middle class/ Upper middle class/ Higher class.

Knowledge about Dengue Fever:

11. Have you ever heard about Dengue fever?
    (i) Yes (ii) No
12. What is the cause of Dengue fever?
    (i) Mosquito's bite (ii) Dirty drinking water (iii) Contaminated food
    (iv) Others (v) Don't know
13. What type of mosquito is carrier of Dengue fever?
    (i) Aedes (ii) Anopheles (iii) All types of mosquito (iv) Don’t know
14. At what time does the Dengue causing mosquito most likely bite?
    (i) Sunrise/sunset (ii) Night (iii) Afternoon (iv) Don’t know
15. During which part of the year is Dengue most prevalent?
    (i) Summers (ii) Rainy season
    (iii) Autumn (iv) Winters
    (v) Don't know
16. Is Dengue Transmissible?
    (i) Yes (ii) No
17. How Dengue is transmitted?
    (i) Human to human contact (ii) Blood transfusion
    (iii) Needle stick injury (iv) Sharing of food/ clothes with the patient
    (v) Don’t know
18. Where Aedes mosquito is breeding?
    (i) In clean water (ii) In unclean water (iii) Don’t know

19. Does Dengue fever viral or Bacterial Disease?

(i) Yes (ii) No (iii) Don’t know

20. What are the signs and symptoms of Dengue fever?

High fever/ Severe body aches/ Nausea and vomiting/ Red spots on the body/ Diarrhoea/ Pain abdomen/ All of the above/ Don’t know

Awareness about Dengue Fever:

21. Do you aware about Dengue fever?

(i) Yes (ii) No

22. Do you feel that you are at risk of getting Dengue fever?

(i) Absolutely (ii) Maybe

(iii) No, I take all the precautions (iv) Don’t know

23. Do you know Aedes mosquito?

(i) Yes (ii) No

24.What do you do initially after noticing any symptoms of fever?

(i) Taking medicine without prescription

(ii) Taking medicine with prescription

(iii) Wait few more days to observe health condition

(iv) Do nothing

25. How do you feel generally after noticing you have infected with fever in the Dengue most preventing time?

(i) Getting very scared (ii) Take it seriously

(iii) Take it normally (iv) Not scared at all (v) No experience

Knowledge about Dengue Fever Treatment:

26. Do you think Dengue fever is treatable?

(i) Yes (ii) No (iii) Don’t know

27. Do you know about primary treatment of Dengue fever without taking any medicine?

(i) Yes (ii) No

28. Do you think does a patient of Dengue require hospitalization?

Definitely/ Sometimes/ No, he can be treated at home/ No treatment required/ Don’t know

29. Do you know which tests are required to diagnose Dengue fever?

(i) Yes (ii) No

30. Do you think Dengue fever is death leading disease without treatment?

(i) Yes (ii) No (iii) Don’t know

31. When do you test after suffering from fever?

(i) Immediately (ii) After getting serious condition (iii) After a few days

(iv) Never do test (v) Didn't face yet

Knowledge & Awareness about Dengue Fever Prevention:

32. Do you think Dengue prevention is possible?

(i) Yes (ii) No (iii) Don’t know

33. Do you know the preventing ways of Dengue fever?

(i) Yes (ii) No

34. Do you think Dengue fever is curable without any treatment?

(i) Yes (ii) No (iii) Don’t know

35. Did/Do you take any step to prevent the reproduction of Aedes mosquito?

(i) Yes (ii) No

36. What are the following options you do to protect yourself against bite of mosquito?

(i) Use Mosquito Spray
(ii) Use Mosquito Coil
(iii) Use Mosquito Repellant/Cream
(iv) Keep closed Windows & Doors
(v) Use Smoke to drive away mosquitoes
(vi) Keep neat & clean my surroundings
(vii) Use Mosquito Net
(viii) Cover my body with long clothes
(ix) Do nothing

37. Did/Do you ever take any step to have uninfected from Dengue fever?

(i) Yes (ii) No

38. Did/Do you ever share your known Dengue preventing information to others for protecting themselves from dengue fever?

(i) Yes (ii) No

39. Where do you get information about Dengue fever prevention from?

(i) TV/radio
(ii) Health care professionals
(iii) Friends and family
(iv) Schools/ Colleges/ Universities
(v) Advertisements/ Billboards/ Banners
(vi) Newspapers/Social media
(vii) Others
(viii) Didn't get yet
